# Suppression of Allelic Recombination and Aneuploidy by Cohesin Is Independent of Chk1 in *Saccharomyces cerevisiae*

**DOI:** 10.1371/journal.pone.0113435

**Published:** 2014-12-31

**Authors:** Shay Covo, Eric Chiou, Dmitry A. Gordenin, Michael A. Resnick

**Affiliations:** Laboratory of Molecular Genetics, National Institute of Environmental Health Sciences, Research Triangle Park, North Carolina, United States of America; National Cancer Institute, United States of America

## Abstract

Sister chromatid cohesion (SCC), which is established during DNA replication, ensures genome stability. Establishment of SCC is inhibited in G2. However, this inhibition is relived and SCC is established as a response to DNA damage, a process known as Damage Induced Cohesion (DIC). In yeast, Chk1, which is a kinase that functions in DNA damage signal transduction, is considered an activator of SCC through DIC. Nonetheless, here we show that, unlike SCC mutations, loss of *CHK1* did not increase spontaneous or damage-induced allelic recombination or aneuploidy. We suggest that Chk1 has a redundant role in the control of DIC or that DIC is redundant for maintaining genome stability.

## Introduction

Chk1 is a kinase in DNA damage signal transduction [1], [2]. Although *chk1* null cells are only modestly sensitive to DNA damage, Chk1 is important for the activation of the G2/M DNA damage response. Chk1 activity stabilizes the checkpoint protein Pds1, preventing premature degradation of the sister chromatid cohesion complex and inappropriate separation of sister chromatids [3]. While Pds1 is normally degraded during anaphase, it is stabilized upon phosphorylation by Chk1 in response to DNA damage.

Although the ability to establish sister chromatid cohesion (SCC) occurs in S phase (for review see [4], [5]), SCC can be also established in response to DNA damage outside of S phase at the site of DNA damage or even across the genome [6]–[12]. Genome-wide DNA damage-induced cohesion is mediated by phosphorylation of the cohesin subunit Mcd1 by Chk1 [13].

We have shown that several hypomorphic mutations in cohesin (the sister chromatid cohesion complex) increase the rate of allelic recombination and chromosome gain [14]–[16]. However, the functional role of Chk1-mediated phosphorylation of cohesin remains to be determined. The *chk1*Δ mutant does not show a reduction in double-strand break repair or damage-induced homologous recombination between sister chromatids [13], [17], [18]. Here, we examined the consequences of *chk1*Δ on genome instability, including chromosome loss or gain as well as allelic recombination. There was no effect of this mutation even after the induction of DNA damage, suggesting a redundant role for Chk1 in controlling DNA damage-induced SCC.

## Material and Methods

### Strain construction

#### Gene knockout and mutant creation

Gene inactivation was done by knockout of specific open reading frames using the KanMX cassette from the *Sacchromyces cerevisiae* deletion collection. The primers that were used for *WPL1* knock out were 5' ATGTTTACTTCAGCCCTTTTT 3' and 5' ACGCTAGAAGGCTCATCAAA 3'. The primers for *CHK1* were 5' AACGCCTTCCTTGATGGAAT 3' and 5' GCGACTTGAACGGAATATCTGT 3'. *mcd1-1* strains were created by pop-in/popout of pVG257 [19] and the mutation was verified by sequencing.

#### Construction of strains for loss of heterozygosity/chromosome loss assay

All genomic locations are according to SGD annotations (http://www.yeastgenome.org/cgi-bin/seqTools)

Haploid strains that were used to construct the loss of heterozygosity strains were transformed with the selectable markers (NAT^R^, *URA3*, HygB^R^, *TRP1*) using PCR products with the following primers. Insertion of NAT cassette to chromosome II position 799056 was done using 5' TTTTGTGATCGGTGTGGCGC GATCTGTGAAATTTAAACTTTTTTGCGGGTGAAACCGAGATTTCTGAAACCGTACGCTGCAGGTCGACGGATCCCC 3' and 5' TCTTTCACACCTGTGCAAAATCCAATGGGAGCAATAAATG TAACATATTT TTTTAACATACCACGGTAGGATCGATGAATTCGAGCTCGTTTTCGA3' as template served pAG25. Validation was done by primers 5' GTATTTGTTATTGCAAGTGGCGTCGC 3' and 5' ACAAAAAGCTGCTGCGGCCCTTCTTA 3'. Insertion of *URA3* cassette to chromosome II at position 241458 was by using primers: 5'TTAGTTTTAATGGAAAACAGTTCCTACAAGCGCTATAACATATGAAATATACATTAGTCGAATATTAACTCAGAGCAGATTGTACTGAGAGTGCACC 3' and 5'AATTCACGCATCTTTATAGAGTTTATAATGCAAATCTCCGCGCGGGGTAATTTGAAGTCCAATTTTTCCGACGCATCTGTGCGGTATTTCACACCGC 3' using pRS306 as a template. Validation was done by using 5' GGCTTTATGTTATCTTCACCATCA 3' and 5' GCATTTTTTTCCTACCACATGGC 3'. Insertion of Hyg cassette to chromosome II at position 235197 was done using primers.

5'TTCCGTAAGTAAAACCGTAAACTTGATACGTTTTTTATTTTCTTTATTAATAGTAATACTATACACTGTCCGTACGCTGCAGGTCGACGGATCCCC 3' and 5' TGGGGTTTTA AAGTAGGTCA TATGAGGAAG ACTGGTATGT CTTTTATCTA ACAGTTTTAT AAATAGCGTCATCGATGAATTCGAGCTCGTTTTCGA 3' as template pAG32 was used. Validation was done by 5' GAAGACAAATTGCAAGTATCCG 3' and 5' GGAAAGTACAGAACAAGAGCAAA 3'. Insertion of *TRP1* cassette to chromosome II at position 23490 was done using primers.

5'GGTGTCACTAACGAAAAATCTAAAGTTTCCTGGAGGACTTTTGTCTGGTTCATTAATTCGTCCAGTAGACG-CAGAGCAGATTGTACTGAGAGTGCACC 3' and TCTCCAATATTACTGCAGGTTAGTACATTATTTTTTACTCGCAGTTGCTATTTTGGCTAGAGGCTGCACGG-CGCATCTGTGCGGTATTTCACACCGC 3', pRS316 was used as a template. Validation was done using 5' GTAATCATCATTGTGGTGTGGAGTGG 3' and 5' GTACTACTCGGTCCGGACAGTAGGAT 3'.

Diploid strains to detect chromosome loss and total loss of heterozygosity were created by mating two opposite mating type haploids, which in this background carry a different mutation in the methionine biosynthesis pathway (*met2Δ, met6Δ*). Diploid cells were selected on methionine-less plates and verified for all other genetic markers.

### Determination of the rates for the different genome instability events

General conditions for rate determination of the different genome instability events are described in the following. Experiments were started by patching at least 6 single colonies from each genotype to YPDA rich medium or YPDA including 1 mM methyl methanesulfonate (MMS) followed by incubation over-night in 30°C, including *mcd1-1*temperature sensitive strains (*mcd1-1* strains are grown and maintained at 23°C prior to experiment). Over-night patches were then spread on selective media (5FOA, SC – tyrosine or SC with 0.9 mM CuSO_4_ plates) and diluted samples were spread on synthetic complete (SC) media. To restrict the effect of *mcd1-1* mutation to the growth phase and not to the selection phase, plates were incubated at 23°C. Plates were incubated for 2-4 days.

Chromosome loss and loss of heterozygosity assays: these assays select first for cells that are resistant of 5FOA due to loss of *URA3* gene function, the rate of this event is calculated as total LOH. The 5FOA resistant colonies were then spotted to YPDA plates and analyzed for loss of the HYG^R^, NAT^R^ and *TRP1* markers, if all are lost then it is considered as chromosome loss. The rate of chromosome loss events (5FOA^R^ ( =  *ura3)*, hyg^s^, nat^s^, *trp1*) were determined.

Chromosome gain assay: The chromosome gain rate was determined from the number of colonies arising on copper-containing medium. Resistance is due to an increase in *CUP1* genes in the haploid strain that contains a single copy of the *CUP1* gene at the end of chromosome V and is primarily due to chromosome gain as was shown in [15]. To determine the rate of copper resistance, undiluted cultures of yeast were spread to synthetic complete media containing 0.9 mM copper (CuSO_4_). In parallel, diluted samples were spread to synthetic complete media to determine the amount of cells in each culture. After 3–4 days the number of copper resistant colonies was determined. For each genotype, the copper resistant colonies were replica-plated to another copper containing plate. Nearly all (over 99%) of the copper resistant colonies were able to grow again on copper plates following replica-plating. Details about inter chromosome recombination assay are found in previous work [14]–[16].

### Survival and allelic recombination in G2-arrested cells treated with ionizing radiation

Haploid cells were grown over-night and diluted to fresh media. They were grown for one to three hours, after which nocodazole was added and cells were incubated another three hours to arrest them in G2. Cells were irradiated with IR (see [20]) with the indicated doses and spread over YPDA plates to determine survival. For allelic recombination, diploid cells were grown and arrested in nocodazole. IR-induced *TYR1* recombination was determined as previously described [14], [16].

## Results and Discussion

### Chk1 is not needed for suppression of DNA damage-induced allelic recombination

**Figure 1:**
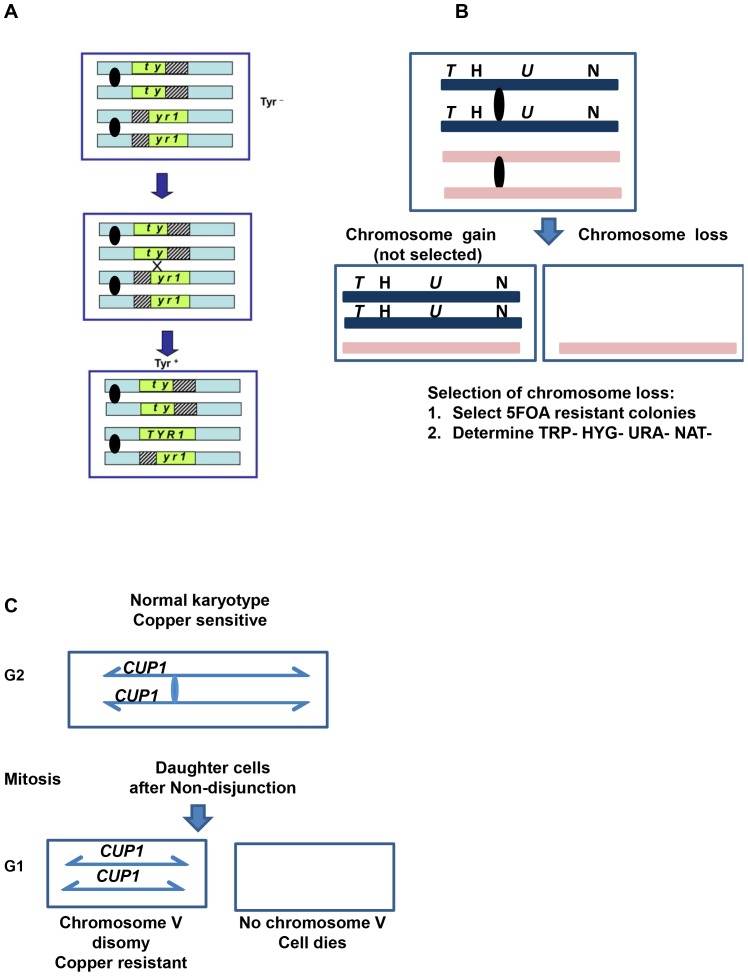
Genetic assays. A. Allelic recombination assay (previously described in [14], [16]). The two homologs of chromosome II in a diploid strain have truncations in the *TYR1* gene with 300 nucleotide overlaps such that recombination generates Tyr+ cells capable of growth on medium lacking tyrosine. B. Chromosome loss and loss of heterozygosity (LOH) assays, described under Material and Methods in "Determination of the rates for the different genome instability events". Briefly, the assay selects first for cells that are resistant of 5FOA due to loss of *URA3* gene function, primarily from LOH. The 5FOA resistant colonies were then analyzed for loss of the hyg, nat and *TRP1* markers. C. Chromosome gain assay (described in details under Materials and Methods "Determination of the rates for the different genome instability events"). The chromosome gain rate was determined from the number of colonies arising on copper-containing medium. Resistance is due to an increase in *CUP1* copies which is primarily due to chromosome gain as was shown in [15].

We have employed genetic reporters to examine whether cells lacking Chk1 have a similar phenotype to sister chromatid cohesion mutants (the strains that were used are presented in Table 1, raw data is found in S1 File). As described in Fig. 1A, we compared the rates of allelic recombination between homologous chromosomes in cells that are WT, defective in SCC (*mcd1-1*), and lacking Chk1 *(chk1Δ)* using an assay that is sensitive to DNA damage-induced recombination (Fig. 1 A and [14], [16]). The *mcd1-1* mutant of the cohesin subunit is temperature-sensitive for growth and genome instability at 30°C (see below). While chronic exposure of *mcd1-1* cells at 30°C to the DNA damaging agent MMS caused a high rate of allelic recombination in comparison to WT, there was no increased recombination in Chk1 deficient cells (Table 2). Wpl1 is a regulator of SCC. Cells deleted for *WPL1* are viable but exhibit subtle defects in SCC [21], [22]. Unlike *chk1Δ* cells, MMS greatly increased allelic recombination in *wpl1Δ* cells (Table 2).

**Table 1:** Strains used in this study.

Strain	Used for	Genotype
CS 1061	Chromosome loss and recombination assay - haploid	*MATa*, *ade5-1, his7-2, leu2-3 112, lys2 A14, trp1Δ, ura3Δ, met6Δ, tyr1 300-1359*
CS 1219	Chromosome loss and recombination assay - haploid	*MATα*, ade5-1 his7-2 leu2-3,112, trp1Δ ura3Δ met2Δ tyr1 1-700 TRP1_ ec,URA3_ec, hygB_ ec, NAT^R^ _ec*
CS 1120	Chromosome loss and recombination assay - haploid	As CS1061 but *mcd1-1*
CS 1145	Chromosome loss and recombination assay - haploid	As CS1061 but *wpl1::G418*
CS1152	Chromosome loss and recombination assay - haploid	As CS1061 but *chk1::G418*
CS1234	Chromosome loss and recombination assay - haploid	As CS1219 but *mcd1-1*
CS1221	Chromosome loss and recombination assay - haploid	As CS1219 but *wpl1::G418*
CS1250	Chromosome loss and recombination assay - haploid	As CS1219 but *chk1::G418*
CS2324	Chromosome loss and recombination assay - diploid (WT)	CS1061XCS1219
CS2338	Chromosome loss and recombination assay - diploid (*mcd1-1*)	CS1120XCS1234
CS2328	Chromosome loss and recombination assay - diploid (*wpl1*Δ)	CS1145XCS1221
CS2285	Chromosome loss and recombination assay - diploid (*chk1*Δ)	CS1250XCS1152
CS1131	Chromosome gain (WT)	*MATa*,*bar1-*Δ, *his7-2*, *trp1Δ*, *ura3Δ*, leu2-3,112,ade2Δ, sfa1Δ, lys2Δ, cup1-1Δ, yhr054cΔ, cup1-2Δ. LYS2*ec, CUP1-1*ec, ADE2*ec, SFA1*ec [25]
CS1143	Chromosome gain haploid	As CS1131 but *mcd1-1*
CS1152	Chromosome gain haploid	As CS1131 but *wpl1*::G418
CS1157	Chromosome gain haploid	As CS1131 but *chk1*::G418

Strains that were used in this study. *ec = ectopic – a genetic marker that was placed not at the native locus (See Material and Methods, under construction of Chromosome Loss assay and below). Strain CS1131 was previously published under different name (TD1 [25]). In this strain several insertion had occurred on chromosome V: *LYS2* gene was inserted at 34,211 bp, *ADE2* at 34,212, *CUP1* and *SFA1* were inserted one next to the other between 36,396 bp and 36,397 bp (all coordinates here and below are given in accordance with Saccharomyces Genome Database). Recombination assay– an assay to measure allelic recombination by reconstitution the *TYR1* gene as described in Fig. 1 A. LOH assay – an assay to measure *URA3* loss of heterozygosity as described in Fig. 1 B. Chromosome gain assay is based on copper resistance as shown in Fig. 1 C.

**Table 2:** Median rates of genome instability events (/10^7^ cell divisions).

Event	WT	*chk1Δ*	*mcd1-1*	*wpl1Δ*
**Allelic recombination**				
MMS	20 (20–40)^18^	33(30–36)^6^	200 (188–450)^9^	100 (95–159)^9^
**Total ** ***URA3*** ** LOH**				
Spontaneous	20 (11–43)^9^	31 (10–40)^6^	5534(3271–7705)^6^	122(76–311)^6^
MMS	103 (62–147)^ 6^	67 (56–81) ^6^	ND	ND
**Chromosome loss** (measured by the number of colonies that acquired 5FOA, hyg, nat, *trp1*)				
Spontaneous	5 (1–8)^6^	<0.5*(<0.5*2)^6^	5531 (3271–7705) ^6^	107 (77–311)^6^
MMS	7 (2–10)^6^	1 (<0.5*–9)^6^	ND	ND
**Chromosome gain** (measured by the number of copper resistant colonies)				
Spontaneous	2 (0.5–2)^21^	2 (0.5–3)^12^	169 (55-254)^9^	1 (0.7–1)^11^
MMS	19 (15–66)^6^	15 (8–30)^6^	ND	ND

The median rate (95% confidence of intervals) of the different genome instability events was measured as described in the text and in Fig. 1; the numbers in superscript (for example ^6^) correspond to the number of repeats. The MMS concentration was 1 mM as described under Material and methods. Allelic recombination, total LOH and chromosome loss were measured in diploid cells; chromosome gain was measured in haploid cells. The rates in the *wpl1* mutants was previously found to not differ statistically from WT (also found there the rate for *mcd1-1* haploid cells [15]). ND – not determined. * indicates a rate lower than indicated, a rate of 5 events per 10^8^ cell divisions was a rate that could be accurately measured using this assay.

**Figure 2:**
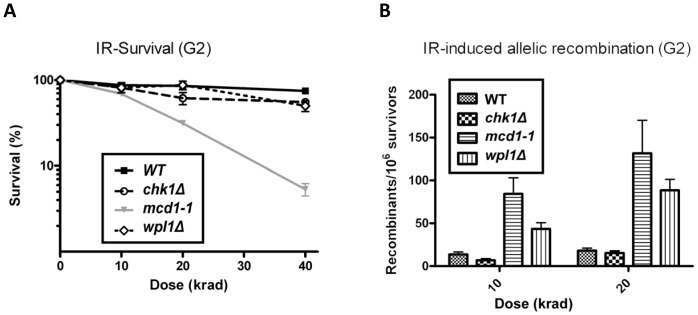
Survival and allelic recombination in G2-arrested cells treated with ionizing radiation. A. G2 arrested haploid cells were irradiated with IR (see [20] and Materials and Methods) with the indicated doses and spread over YPDA plates to determine survival. B. Similarly, G2 arrested diploid cells were irradiated. IR-induced *TYR1* recombination was determined as previously described [14], [16].

Chronic exposure to MMS appears to cause genome instability primarily through interfering with DNA replication [23]. The absence of an effect of the *CHK1* deletion could result if this gene functions primarily in G2 phase of the cell cycle. To study if Chk1 is important for DSB repair or for suppression of allelic recombination when DSBs are induced in the G2 phase of the cell cycle, we exposed nocodazole-arrested haploid cells to ionizing radiation (IR). In contrast to the high sensitivity of *mcd1-1* cells, the *chk1* and *wpl1* deficient cells showed little effect in survival as compared to WT cells (Fig. 2 A). The effect of IR on allelic recombination was measured in diploid cells. Previously, we and others showed that even a modest reduction in the amount of cohesin leads to inefficient DSB repair and an increase in allelic recombination similar to what is found for the *mcd1-1* mutant (Fig. 2 B and [14], [16], [24]). As shown in Fig. 2 B, the subtle SCC defect associated with *wpl1*Δ cells also led to high levels of IR-induced allelic recombination. However, there was little difference in IR-induced allelic recombination between WT and *chk1*Δ cells up to the maximum dose examined (20 krad). The fact that deletion of *CHK1* does not produce the same effect as the cohesin mutants indicates that Chk1 has either a minor or redundant role in the cohesion pathway important for preventing mitotic recombination between homologous chromosomes.

### Chk1 is not needed for suppression of spontaneous or DNA damage-induced chromosome loss

Since defects in SCC are expected to reduce the fidelity of chromatid transmission, the rate of chromosome loss was measured in *chk1Δ* cells using a diploid-based reporter assay. As described in Fig. 1 B, four ectopic markers were inserted into one of the two chromosome II homologs of a diploid strain. Chromosome loss was identified by loss of all four markers. First, the loss of *URA3* was selected as resistance to 5-Fluoroorotic acid (5FOA). Next, the phenotype for the other three marker genes was examined. Chromosome loss is concluded if all four markers are lost. While the rate of chromosome loss in *mcd1-1* and *wpl1Δ* was 100-fold and 20-fold higher than in WT cells, respectively, there was little difference in chromosome loss between WT and *chk1Δ* cells (Table 2). Also, WT and *chk1Δ* cells had similar rates of appearance of 5FOA resistant cells, indicating that there are no major differences in other pathways that might lead to inactivation of the *URA3* gene, such as point mutations or recombination (Table 2). Furthermore, there was no major difference in chromosome loss between the WT and *chk1Δ* for cells chronically exposed to MMS (Table 2). (Due to the nature of 5FOA selection, this assay was conducted under conditions of low, chronic exposure as described under Material and Methods.)

### Chk1 is not needed for suppression of spontaneous or DNA damage-induced chromosome gain

The rates of chromosome gain were also determined since gain provides direct evidence for defects in chromosome transmission. Since Chk1 activates Pds1 and presumably phosphorylates a cohesin subunit (Mcd1), which are major regulators of chromosome transmission, a mutation in *CHK1* might be expected to increase chromosome gain rates [3], [13]. Chromosome gain was measured by increased resistance to copper, which is due to a change in copy number of the metallothionein gene *CUP1*. In our assay that was adapted from [25] and was recently published [15], the *CUP1* gene was inserted into chromosome V (Fig. 1). Recently, we established that all copper resistant colonies acquired resistance through gain of chromosome V [15]. As shown in Table 2, chromosome gain rates are much higher in *mcd1-1* mutants than WT cells (up to 170-fold increase). However, there was no difference between WT and *chk1Δ* strains for cells growing spontaneously or in the presence of low levels of MMS, although MMS did induce increase in both types of cells.

While we could not see any major effect of a *CHK1* deletion in any of our assays, absence of this gene can have genetic consequences. For example, loss of *CHK1* leads to increases in gross chromosomal rearrangements (GCR) at the end of chromosome V [26]. The *CHK1* deletion also resulted in loss of chromosome III in strains where several origin of replication where depleted [26]. Removal of these origins resulted in a long stretch of chromosome lacking an origin, leading to a delay in completion of DNA synthesis. It was proposed that Chk1 may function as a replication stress checkpoint pathway activated by failure of origin firing [27].

In conclusion, our results show that the sub-pathway of damage-induced cohesion controlled by Chk1is not required for the maintenance of several types of genome stability including accurate chromosome distribution and the prevention of recombination between homologous chromosomes. While these findings are unexpected in light of the targets Chk1 is supposed to phosphorylate [3], [13], it is possible that the absence of a role Chk1 in preventing the genome instabilities investigated here might be due to it functioning in a back-up, redundant pathway.

## Supporting Information

S1 File **Raw data of the results presented in** **Fig. 2** **and** **Table 2** **.**
(XLSX)
